# Supplementary material for: Pyruvate Dehydrogenase Kinase 1 inhibition mediated oxidative phosphorylation enhancement in cartilage promotes osteoarthritis progression
Source: BMC Musculoskelet Disord. 2023 Jul 20;24:597. doi: 10.1186/s12891-023-06585-6 (PMC10357736; doi:10.1186/s12891-023-06585-6)
Supplement: Supplementary file 1 — Additional file 1. [file 12891_2023_6585_MOESM1_ESM.zip › BP.Gsea.1653623667859/GOBP_P38MAPK_CASCADE.html]

Details for gene set GOBP\_P38MAPK\_CASCADE[GSEA]

|  || Dataset | 55457\_collapsed\_to\_symbols.cls.cls #OA\_versus\_normal.cls.cls #OA\_versus\_normal\_repos |
| Phenotype | cls.cls#OA\_versus\_normal\_repos |
| Upregulated in class | normal |
| GeneSet | GOBP\_P38MAPK\_CASCADE |
| Enrichment Score (ES) | -0.4565557 |
| Normalized Enrichment Score (NES) | -1.7640624 |
| Nominal p-value | 0.0016891892 |
| FDR q-value | 0.115314655 |
| FWER p-Value | 0.998 |
Table: GSEA Results Summary

  

Fig 1: Enrichment plot: GOBP\_P38MAPK\_CASCADE      
 Profile of the Running ES Score & Positions of GeneSet Members on the Rank Ordered List

  

| SYMBOL | TITLE | RANK IN GENE LIST | RANK METRIC SCORE | RUNNING ES | CORE ENRICHMENT || 1 | TREM2 | triggering receptor expressed on myeloid cells 2 [Source:HGNC Symbol;Acc:HGNC:17761] | 342 | 0.726 | 0.0119 | No |
| 2 | PTPN22 | protein tyrosine phosphatase non-receptor type 22 [Source:HGNC Symbol;Acc:HGNC:9652] | 578 | 0.630 | 0.0271 | No |
| 3 | MAP3K7 | mitogen-activated protein kinase kinase kinase 7 [Source:HGNC Symbol;Acc:HGNC:6859] | 1701 | 0.399 | -0.0391 | No |
| 4 | PJA2 | praja ring finger ubiquitin ligase 2 [Source:HGNC Symbol;Acc:HGNC:17481] | 1898 | 0.373 | -0.0345 | No |
| 5 | NCF1 | neutrophil cytosolic factor 1 [Source:HGNC Symbol;Acc:HGNC:7660] | 2195 | 0.339 | -0.0396 | No |
| 6 | MAPK14 | mitogen-activated protein kinase 14 [Source:HGNC Symbol;Acc:HGNC:6876] | 2352 | 0.320 | -0.0347 | No |
| 7 | HGF | hepatocyte growth factor [Source:HGNC Symbol;Acc:HGNC:4893] | 2547 | 0.297 | -0.0340 | No |
| 8 | EZR | ezrin [Source:HGNC Symbol;Acc:HGNC:12691] | 3817 | 0.174 | -0.1235 | No |
| 9 | GADD45A | growth arrest and DNA damage inducible alpha [Source:HGNC Symbol;Acc:HGNC:4095] | 4081 | 0.148 | -0.1362 | No |
| 10 | LGALS9 | galectin 9 [Source:HGNC Symbol;Acc:HGNC:6570] | 4670 | 0.098 | -0.1767 | No |
| 11 | CYLD | CYLD lysine 63 deubiquitinase [Source:HGNC Symbol;Acc:HGNC:2584] | 5664 | 0.018 | -0.2530 | No |
| 12 | DLG1 | discs large MAGUK scaffold protein 1 [Source:HGNC Symbol;Acc:HGNC:2900] | 6219 | -0.022 | -0.2949 | No |
| 13 | PRMT1 | protein arginine methyltransferase 1 [Source:HGNC Symbol;Acc:HGNC:5187] | 6319 | -0.029 | -0.3011 | No |
| 14 | MAP2K6 | mitogen-activated protein kinase kinase 6 [Source:HGNC Symbol;Acc:HGNC:6846] | 7246 | -0.099 | -0.3679 | No |
| 15 | SASH1 | SAM and SH3 domain containing 1 [Source:HGNC Symbol;Acc:HGNC:19182] | 7284 | -0.101 | -0.3654 | No |
| 16 | PHLPP1 | PH domain and leucine rich repeat protein phosphatase 1 [Source:HGNC Symbol;Acc:HGNC:20610] | 7465 | -0.115 | -0.3733 | No |
| 17 | MINK1 | misshapen like kinase 1 [Source:HGNC Symbol;Acc:HGNC:17565] | 7488 | -0.117 | -0.3687 | No |
| 18 | DUSP10 | dual specificity phosphatase 10 [Source:HGNC Symbol;Acc:HGNC:3065] | 7775 | -0.140 | -0.3836 | No |
| 19 | BMP4 | bone morphogenetic protein 4 [Source:HGNC Symbol;Acc:HGNC:1071] | 7816 | -0.142 | -0.3792 | No |
| 20 | HAND2 | heart and neural crest derivatives expressed 2 [Source:HGNC Symbol;Acc:HGNC:4808] | 8211 | -0.172 | -0.4007 | No |
| 21 | RASGRP1 | RAS guanyl releasing protein 1 [Source:HGNC Symbol;Acc:HGNC:9878] | 8352 | -0.182 | -0.4019 | No |
| 22 | XDH | xanthine dehydrogenase [Source:HGNC Symbol;Acc:HGNC:12805] | 8366 | -0.183 | -0.3932 | No |
| 23 | AGER | advanced glycosylation end-product specific receptor [Source:HGNC Symbol;Acc:HGNC:320] | 8562 | -0.198 | -0.3978 | No |
| 24 | ATF2 | activating transcription factor 2 [Source:HGNC Symbol;Acc:HGNC:784] | 8592 | -0.201 | -0.3894 | No |
| 25 | MAP3K4 | mitogen-activated protein kinase kinase kinase 4 [Source:HGNC Symbol;Acc:HGNC:6856] | 8637 | -0.203 | -0.3821 | No |
| 26 | GADD45G | growth arrest and DNA damage inducible gamma [Source:HGNC Symbol;Acc:HGNC:4097] | 8671 | -0.206 | -0.3737 | No |
| 27 | LEP | leptin [Source:HGNC Symbol;Acc:HGNC:6553] | 8740 | -0.212 | -0.3678 | No |
| 28 | CAV3 | caveolin 3 [Source:HGNC Symbol;Acc:HGNC:1529] | 8953 | -0.228 | -0.3722 | No |
| 29 | SPI1 | Spi-1 proto-oncogene [Source:HGNC Symbol;Acc:HGNC:11241] | 9012 | -0.232 | -0.3643 | No |
| 30 | ZC3H12A | zinc finger CCCH-type containing 12A [Source:HGNC Symbol;Acc:HGNC:26259] | 9030 | -0.234 | -0.3532 | No |
| 31 | OPRK1 | opioid receptor kappa 1 [Source:HGNC Symbol;Acc:HGNC:8154] | 9046 | -0.235 | -0.3419 | No |
| 32 | STK39 | serine/threonine kinase 39 [Source:HGNC Symbol;Acc:HGNC:17717] | 9206 | -0.248 | -0.3411 | No |
| 33 | MAP3K3 | mitogen-activated protein kinase kinase kinase 3 [Source:HGNC Symbol;Acc:HGNC:6855] | 10121 | -0.333 | -0.3945 | No |
| 34 | BMP2 | bone morphogenetic protein 2 [Source:HGNC Symbol;Acc:HGNC:1069] | 10919 | -0.420 | -0.4343 | Yes |
| 35 | MAP3K5 | mitogen-activated protein kinase kinase kinase 5 [Source:HGNC Symbol;Acc:HGNC:6857] | 10923 | -0.421 | -0.4122 | Yes |
| 36 | MAP3K20 | mitogen-activated protein kinase kinase kinase 20 [Source:HGNC Symbol;Acc:HGNC:17797] | 11475 | -0.498 | -0.4286 | Yes |
| 37 | EMC10 | ER membrane protein complex subunit 10 [Source:HGNC Symbol;Acc:HGNC:27609] | 11497 | -0.501 | -0.4037 | Yes |
| 38 | MAPK11 | mitogen-activated protein kinase 11 [Source:HGNC Symbol;Acc:HGNC:6873] | 11501 | -0.501 | -0.3773 | Yes |
| 39 | MFHAS1 | multifunctional ROCO family signaling regulator 1 [Source:HGNC Symbol;Acc:HGNC:16982] | 11898 | -0.571 | -0.3778 | Yes |
| 40 | MAP2K3 | mitogen-activated protein kinase kinase 3 [Source:HGNC Symbol;Acc:HGNC:6843] | 12004 | -0.593 | -0.3545 | Yes |
| 41 | MAPKAPK2 | MAPK activated protein kinase 2 [Source:HGNC Symbol;Acc:HGNC:6887] | 12094 | -0.619 | -0.3286 | Yes |
| 42 | IL1B | interleukin 1 beta [Source:HGNC Symbol;Acc:HGNC:5992] | 12105 | -0.622 | -0.2964 | Yes |
| 43 | SPHK1 | sphingosine kinase 1 [Source:HGNC Symbol;Acc:HGNC:11240] | 12320 | -0.682 | -0.2768 | Yes |
| 44 | PER1 | period circadian regulator 1 [Source:HGNC Symbol;Acc:HGNC:8845] | 12496 | -0.746 | -0.2508 | Yes |
| 45 | VEGFA | vascular endothelial growth factor A [Source:HGNC Symbol;Acc:HGNC:12680] | 12629 | -0.813 | -0.2180 | Yes |
| 46 | GADD45B | growth arrest and DNA damage inducible beta [Source:HGNC Symbol;Acc:HGNC:4096] | 12816 | -1.001 | -0.1793 | Yes |
| 47 | ZFP36 | ZFP36 ring finger protein [Source:HGNC Symbol;Acc:HGNC:12862] | 12846 | -1.052 | -0.1257 | Yes |
| 48 | DUSP1 | dual specificity phosphatase 1 [Source:HGNC Symbol;Acc:HGNC:3064] | 12884 | -1.202 | -0.0648 | Yes |
| 49 | ZFP36L1 | ZFP36 ring finger protein like 1 [Source:HGNC Symbol;Acc:HGNC:1107] | 12888 | -1.244 | 0.0009 | Yes |
Table: GSEA details [plain text format]

  

Fig 2: GOBP\_P38MAPK\_CASCADE      
 Blue-Pink O' Gram in the Space of the Analyzed GeneSet

  

Fig 3: GOBP\_P38MAPK\_CASCADE: Random ES distribution      
 Gene set null distribution of ES for **GOBP\_P38MAPK\_CASCADE**
